# General practitioner experiences and perceptions of the ANZAED Eating Disorder Credential

**DOI:** 10.1186/s40337-026-01622-w

**Published:** 2026-05-19

**Authors:** Kristy Green, Phillipa Hay, Janet Conti, Jade Gooding, Siân A. McLean, Gabriella Heruc

**Affiliations:** 1 School of Medicine, Western Sydney University, Sydney, Australia; 2 Translational Health Research Institute, School of Medicine, Western Sydney University, Sydney, Australia; 3 Mental Health Services, Campbelltown Hospital, Campbelltown, Australia; 4 School of Psychology, Western Sydney University, Sydney, Australia; 5 Australia & New Zealand Academy for Eating Disorders, Sydney, Australia; 6 School of Psychology and Public Health, La Trobe University, Melbourne, Australia; 7 Eating Disorders and Nutrition Research Group, Translational Health Research Institute, School of Medicine, Western Sydney University, Locked Bag 1797, Penrith, Sydney, 2751 Australia

## Abstract

**Background:**

The Australia & New Zealand Academy for Eating Disorders (ANZAED) established a credentialing system for general practitioners (GPs) in 2024. The ANZAED Eating Disorder Credential formally recognises GPs who have the necessary knowledge and training to provide safe and effective care to patients with eating disorders (EDs). The aim of this study was to learn about the experiences of both credentialed and non-credentialed GPs, with a particular focus on their reasons for becoming credentialed, barriers to becoming credentialed, and how the Credential has impacted their clinical practice.

**Methods:**

Thirty-eight GPs (37 female, 21 credentialed, 17 non-credentialed) completed an online mixed-methods survey, with both open-ended questions and multiple-choice questions. The survey asked about their perceptions of the Credential and their experiences with the credentialing system.

**Results:**

The main motivations to become credentialed were to receive recognition for their training (n = 15, 71.4%) and improve their knowledge about EDs (n = 9, 42.9%). The main reasons to not become credentialed were not wanting to increase their ED caseload (n = 11, 64.7%) or be identified as an ED specific GP (n = 10, 58.8%). Three quarters (n = 16, 76.2%) of credentialed GPs did not perceive their ED patient caseload changing after becoming credentialed. Content analysis of open-ended questions yielded two themes: (1) The value of the Credential; and (2) Need for increased clarity about the Credential. Theme 1 highlighted the financial and workload barriers to becoming credentialed, whilst also indicating that GPs perceived that the Credential would improve their care of EDs. Theme 2 identified some areas where there was a need for increased understanding of the Credential’s requirements for GPs and its potential impact on people living with EDs.

**Conclusions:**

GPs are motivated to become credentialed to gain recognition for their ED knowledge and training. However, improvements in the credentialing system may encourage its uptake. These may include increasing the Credential’s visibility and recognition, addressing perceptions of the financial impact, improving clarity about the Credential’s overall aims and providing additional support to GPs in their learning.

**Supplementary Information:**

The online version contains supplementary material available at 10.1186/s40337-026-01622-w.

## Abstract (plain language summary)

The ANZAED Eating Disorder Credential for general practitioners (GPs) was introduced to recognise GPs with additional training and knowledge to care for people with eating disorders. GPs who became credentialed mainly did so to gain formal recognition for their eating disorder training and to improve their knowledge, while those who did not become credentialed often worried about increased workload or being identified as an eating disorder specialist GP. Most GPs who were credentialed did not report seeing more eating disorder patients after receiving the Credential, suggesting concerns about increased caseload may not be realised in practice. GPs generally saw value in the Credential but identified a need for clearer information about its purpose, requirements, and impact, as well as concerns about cost and workload.

**Supplementary Information:**

The online version contains supplementary material available at 10.1186/s40337-026-01622-w.

## Introduction

Eating disorders (EDs) are serious mental health conditions that sometimes, but not always, require complex management. They are characterised by the disruption of eating behaviours that compromise health and psychosocial wellbeing [1]. In 2023, 4.5% of all Australians had an ED, with a lifetime prevalence of 10.5% [2]. Of these individuals, only half reach full recovery [3], with 1273 deaths in Australia in 2023 attributed to EDs [2]. Given the complexity and seriousness of EDs, there is a need for health care providers to be competent at screening, diagnosis and management of EDs to improve outcomes, particularly in general practice, which is typically the first point of contact with health services for people with EDs [4]. To improve knowledge and confidence in general practitioners (GPs; also known as family physicians) and help people with EDs access safe and effective care, the Australia & New Zealand Academy for Eating Disorders (ANZAED) recently developed an ED Credential for GPs, which formally recognises their knowledge and completion of additional training in the screening, assessment, diagnosis and referral for treatment of EDs. The aim of this study was to explore GPs’ experiences and perceptions of the newly developed ANZAED credentialing system.

There is an urgent need to help build GPs’ skills and knowledge in the management of EDs, with a survey in 2023 revealing only 15% of Australian GPs or family physicians feel equipped to manage their patients with EDs [5]. Adding to this, GPs are often held responsible for barriers experienced accessing care [6], with people living with EDs and their families struggling to navigate the healthcare system and find adequate care [7, 8]. A recent systematic review found that people living with an ED, on disclosure of ED symptoms, reported often feeling invalidated by their GP [9]. These experiences were described as contributing to feelings of shame and were identified as a barrier to seeking further treatment [9]. Similarly, a qualitative study showed that parents of children with EDs felt GPs downplayed the severity of their child’s symptoms [8]. It has also been found that a notable proportion of GPs hold negative views and misconceptions towards EDs [10]. This includes using low BMI as the primary indicator of ED severity [9], believing that EDs only affect women [9] and that EDs are self-inflicted [10]. Furthermore, a recent qualitative study reported that some people living with an ED experienced their GPs as being inadequately trained to provide appropriate screening, assessment and onward referral. This, they felt, contributed to delays in treatment [11]. These aforementioned factors risk weakening the therapeutic alliance between the patient and GP and have been perceived by people living with an ED to contribute to increased rates of relapse and ambivalence towards treatment [9].

It has been suggested that insufficient knowledge and training may affect GPs’ provision of ED care. This insufficient training may result from the availability of time and space for ED education within GP curricula. In Australia, the most common training pathway for GPs is a postgraduate course provided by the Royal Australian College of General Practitioners (RACGP), which provides teaching about EDs as a subsection within larger topics, however, it is not considered to be core content [12]. Despite the limited time given to ED education, tertiary educators highly value ED training and believe it is important [12]. The lack of attention to ED education is caused by barriers faced by tertiary education providers, such as having an already full curriculum, cost constraints, insufficient access to current evidence, and limited opportunity for students to practice skills [12]. Hence, the limited amount of training GPs receive could explain why they feel unequipped to manage EDs [5] and why ED patients may have negative experiences when seeking treatment [9].

In 2020, ANZAED created a credentialing system for ED clinicians, including dietitians and mental health professionals (MHPs) [13], based on ANZAED’s clinical practice and training standards [14]. GPs were eligible to apply for the MHP Credential if they were a provider of focused psychological strategies (FPS), recognising that this Credential pathway reflects provision of psychological treatment for EDs [15]. To qualify for the MHP Credential, all MHPs, including GPs, are required to complete both introductory and mental health treatment provision training and annually undertake 15 h of continuing professional development (CPD) and 6 h of clinical supervision relevant to EDs, allowing consultation between a clinician and their supervisor about their practice [15]. Since its introduction, only 11 GPs have received the ANZAED MHP credential.

Whilst most GPs are not direct providers of FPS [16], they have a very important role in the care of ED patients. Seeing a GP is often the first step towards assessment, diagnosis referral, and ongoing medical care for people with an ED who are seeking help. In Australia this referral may include the GP initiating an Eating Disorder Treatment Plan to subsidise sessions with a MHP and dietitian. They therefore need to be competent at supporting their patients, medically and emotionally, whilst coordinating and awaiting access for ED multidisciplinary treatment. To address the need to support development and recognition of competence, ANZAED expanded the Credential in 2024 to create a new Credential pathway specifically for GPs, recognising their ability to assess, diagnose and refer to appropriate treatment. They can then create a profile in the searchable online connect·ed directory which is available to the public, both those with lived experience and referrers, to find suitably qualified and trained health professionals [17, 18]. This visibility is particularly important to people seeking treatment for an ED. Although GPs are generalist practitioners with training in mental and physical health conditions, identification of those GPs with particular interest and additional training specifically in ED management, provides greater confidence to people living with EDs and their carers in seeking out a health professional. In regard to training, to become credentialed in the GP pathway, GPs must complete one of two introductory training modules, however there are no ongoing professional development or supervision requirements [17]. Additionally, there is an initial application fee of $100 and an annual fee of $170. Within three months, from May to July 2024, 51 GPs became credentialed.

Given the large GP workforce in Australia, and the comparatively small number who have become credentialed thus far, it would be helpful to understand GPs’ perceived drivers and deterrents of becoming credentialed. Although the additional training required by the Credential can help update knowledge and skill, not all GPs will voluntarily seek out additional training, depending on perceived costs, convenience and relevance [19]. For dietitians and MHPs, the Credential reportedly increased their confidence and willingness to deliver ED care [20], which may be a similar consequence of the GP Credential. Understanding GPs’ views of credentialing will inform the ongoing operation of the Credential and potential for the Credential to enhance ED clinical care within general practice.

This study therefore aimed to survey both credentialed and non-credentialed GPs about their experience with the Credential. The survey explored their views on the Credential, reasons to become or not become credentialed, and the impact of the Credential on clinical practice.

## Methods

### Design and procedure

This study used a mixed methods design with data collection via a self-report survey and was approved by Western Sydney University Human Research Ethics Committee (HREC Approval Number: H15252). The study was advertised to potential participants by stakeholder organisations on social media and via direct email to GPs who had registered with ANZAED to learn more about the GP Credential. From June 2025 to July 2025, GPs completed an online survey via the Qualtrics platform. Thirty-eight participants gave informed consent and participated in the study. Participants were included in the study if they consented and confirmed they were a GP fully registered with the Australian Health Practitioner Regulation Agency (AHPRA). To maintain confidentiality, participants were asked to create a unique ID. The survey took about 10 min to finish. Participants received no compensation for completing the survey.

### Measures

The survey had a total of 40 items (35 closed-response questions and five open-ended, free text questions) and consisted of three parts: (i) demographics; (ii) professional and ED treatment experience and training (e.g,. percentage of patients seen with EDs, age groups, and types of ED presentations seen); and (iii) the ANZAED Eating Disorder Credential section, which included questions on their views of the Credential, use of the connect·ed website, and recommendations for the Credential. Open-text questions invited responses to questions about motivations to become credentialed, benefits of the Credential, use of the connect·ed searchable directory to create a profile, and recommendations to improve the Credential (overall and in relation to the searchable directory). Responses to closed-response questions about the ease of the Credential application [“*Please rate the ease of the Credential’s application process*”] and whether becoming a credentialed MHP and/or GP impacted the proportion of ED patients in their caseload [“*To what extent has attaining the ANZAED Eating Disorder Credential changed the number of eating disorder patients you treat on a weekly basis?*”] were rank ordered using ordinal scales.

### Data analysis

Quantitative analyses were conducted using Microsoft Excel software (Version 16.98) and IBM SPSS Statistics 30. Categorical and discrete data were presented as frequency tables. Continuous numerical data were summarised using descriptive statistics. Data were inspected for normality using quantile plots and the Shapiro–Wilk test. If normally distributed, they were summarised by the mean and standard error of mean (SEM). If not normally distributed, they were summarised by the median and interquartile range (IQR).

An inductive content analysis [21] of the responses to the five open-ended questions was performed with responses housed on Microsoft Excel spreadsheet software (Version 16.98). Two authors (KG and GH) individually read participant responses, assigned codes and derived themes. They met multiple times throughout the process to discuss their findings and create a joint understanding, which allowed them to reach an agreement on the draft themes and subthemes. All authors reviewed and agreed upon the final themes and subthemes.

### Reflexivity

KG is a White cis-gender female and a fourth-year medical student at Western Sydney University. GH is a White cis-gender female and an Accredited Practising Dietitian, the former Head of Credentialing for ANZAED, a Credentialed Eating Disorder Clinician and a researcher in EDs. PH is a White cis-gender female and a Psychiatrist, Credentialed Eating Disorder Clinician and researcher in EDs. JC is a White cis-gender female and a Clinical Psychologist and Credentialed Eating Disorder Clinician. JG is a White cis-gender female and Clinical Psychologist and the Chief Executive Officer of ANZAED. SM is a White, cis-gender female and psychology academic and Chair of the ANZAED Credentialing Governing Council. The team’s professional experience in ED care and credentialing brings valuable insight but may also influence interpretations of the analysis.

## Results

### Participant characteristics

Thirty-eight GPs (median age: 43, IQR: 19) completed the survey. Most were women (97.4%) and identified as Oceanian (44.7%) or European (42.1%). All GPs resided in Australia, and most GPs reported that they practiced in New South Wales (36.8%), Queensland (23.7%), Western Australia (15.8%) or Victoria (13.2%). This reflects the general population as these are the four most populated states in Australia [22]. Approximately one third of GPs (36.9%) reported they primarily worked in regional or rural settings. All the GPs spoke English at work, and 10.5% spoke a second language.

Regarding professional and ED care experience, all GPs reported working in outpatient settings, with most reporting that they worked in a private practice (89.5%). GPs had a median of 13.5 years’ experience (IQR: 15.25) working as a GP and a mean of nine years (IQR: 6.25) experience caring for people with EDs. Most GPs were involved in coordinating care (94.7%) and diagnosing EDs (92.1%). Most GPs (94.7%) reported that less than half of their patients had EDs and that they spent less than 16 h each week seeing patients with EDs. The most common ED presentation seen was anorexia nervosa. Of the GPs who participated in the study, 50.0% were credentialed as GPs, 5.3% were credentialed as MHPs and 44.7% were not credentialed. For further details about participant characteristics, see Table 1.

**Table 1:** Participant demographic characteristics (n = 38)

Clinician characteristic	n	Percent
Gender^a^		
Female	37	97.4
Ethnicity^a,b^		
Oceanian (e.g., Australian, New Zealand, Polynesian)	17	44.7
European (e.g., British, Irish, Western European, Northern European)	16	42.1
Aboriginal or Torres Strait Islander^a^		
No	37	97.4
State/territory of practice^b^		
New South Wales (NSW)	14	36.8
Queensland (QLD)	9	23.7
Victoria (VIC)	5	13.2
Western Australia (WA)	6	15.8
Australian Capital Territory (ACT), Northern Territory (NT), South Australia (SA), Tasmania (TAS)	5	13.2
Geographical area of practice		
Metropolitan	24	63.2
Regional or rural	14	36.8
Workplace service		
Eating disorder specific service	0	0.0
Generalist/broad mental health service	6	15.8
Generalist/broad health service	32	84.2
Proportion of patients seen with EDs		
Few (< 25%)	22	57.9
A substantial number (> 25%)	16	42.1
Hours spent seeing patients with EDs per week		
0–3 h (half a day or less)	19	50.0
4–8 h (1 day or less)	11	28.9
9–16 h (2 days or less)	6	15.8
17–24 h (3 days or less)	2	5.3
Types of care provided^b^		
I provide diagnosis and referral	35	92.1
I coordinate care, including providing medical monitoring	36	94.7
I provide mental health treatment (e.g., focused psychological strategies)	13	34.2
Other	2	5.3
Credentialed Eating Disorder Clinician		
Yes—I am credentialed as a General Practitioner	19	50.0
Yes—I am credentialed as a Mental Health Professional	2	5.3
No, I am not a Credentialed Eating Disorder Clinician	17	44.7
Most common ED seen		
Anorexia nervosa	12	31.5
Binge eating disorder	10	26.3
Atypical anorexia nervosa	8	21.1
Subthreshold binge eating disorder	4	10.5
Avoidant restrictive food intake disorder (ARFID)	3	7.9
Bulimia nervosa	1	2.6

^a^Cells may not add up to 100% to preserve confidentiality of participants

^b^Participants were able to select more than one answer

### Professional development

GPs reported completing a median of 5 h (IQR: 6) of ED professional development each year. Fourteen GPs (36.8%), of whom 10 were credentialed and 4 non-credentialed, indicated that they participate in ED supervision, with a median of 11 h (IQR: 8.25) of completed ED supervision each year (see Table 2). Of those GPs, eight (57.1%) received supervision in the format of group sessions, six (42.9%) attended one-on-one sessions, and four (28.6%) received peer-based supervision, with some participating in more than one type of supervision. Twenty-six GPs (68.4%) indicated they were interested in group supervision, including those already participating in group supervision (see Table 2). Of those interested in supervision, eleven (78.6%) credentialed GPs were willing to pay, however only three (25.0%) non-credentialed GPs were willing to pay for supervision.

**Table 2:** Eating disorder supervision of GPs

Supervision characteristic	Credentialed GPs		Non-credentialed GPs		Total	
	n	Percent	n	Percent	n	Percent
Current participation in ED supervision						
Yes	10	47.6	4	23.5	14	36.8
No	11	52.4	13	76.5	24	63.2
Interest in group ED supervision						
Yes	14	66.7	12	70.6	26	68.4
No	7	33.3	5	29.4	12	31.6
Amount willing to pay for group ED supervision^ab^						
$60–$120	10	71.4	3	25.0	13	50.0
Not willing to pay	3	21.4	9	75.0	12	46.2

^a^Cells may not add up to 100% to preserve confidentiality of participants

^b^Currency is Australian dollars

### Perceptions of the credential

**Fig. 1:**
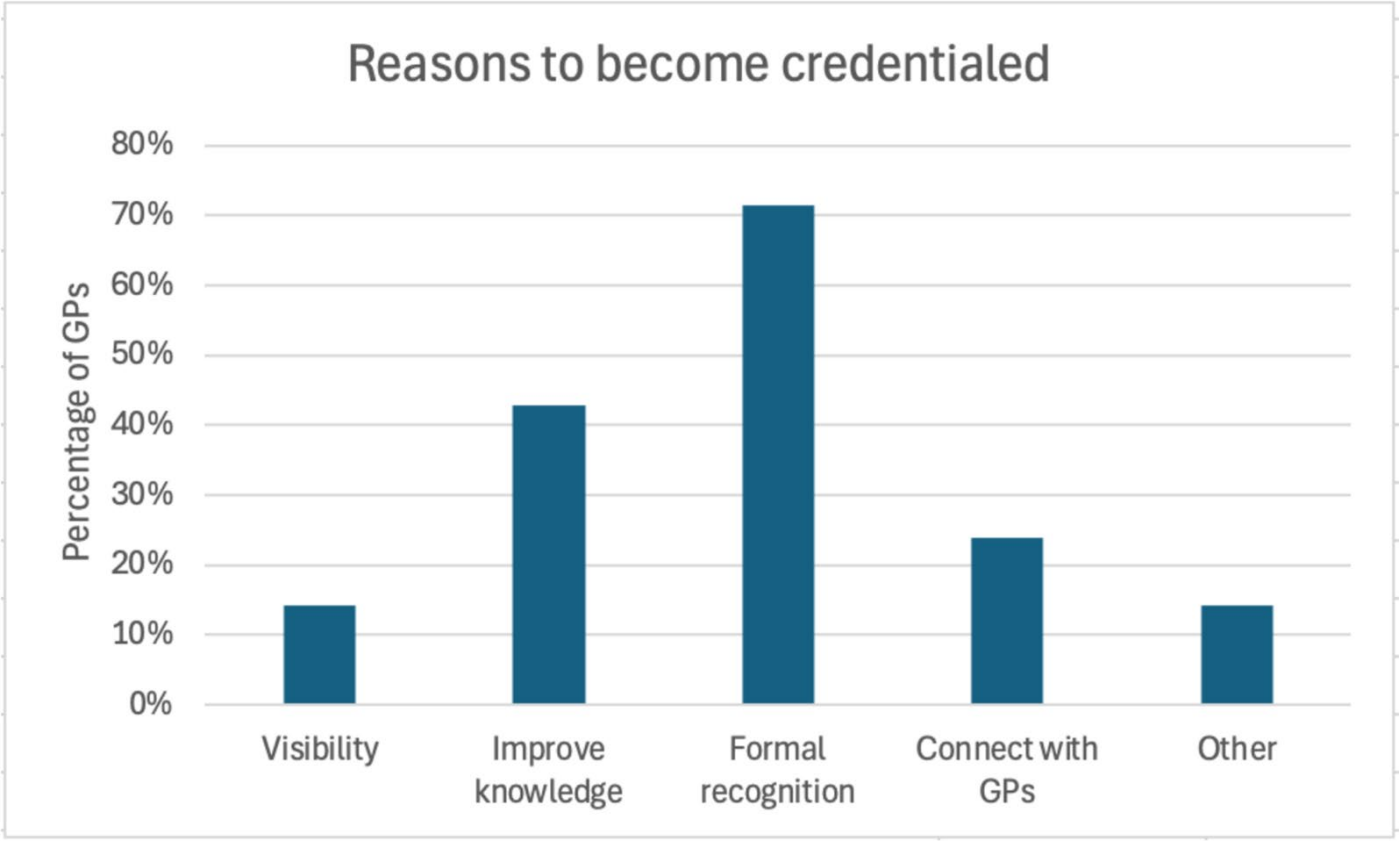
Reasons GPs provided for becoming credentialed*. *Participants were able to select more than one answer

**Fig. 2:**
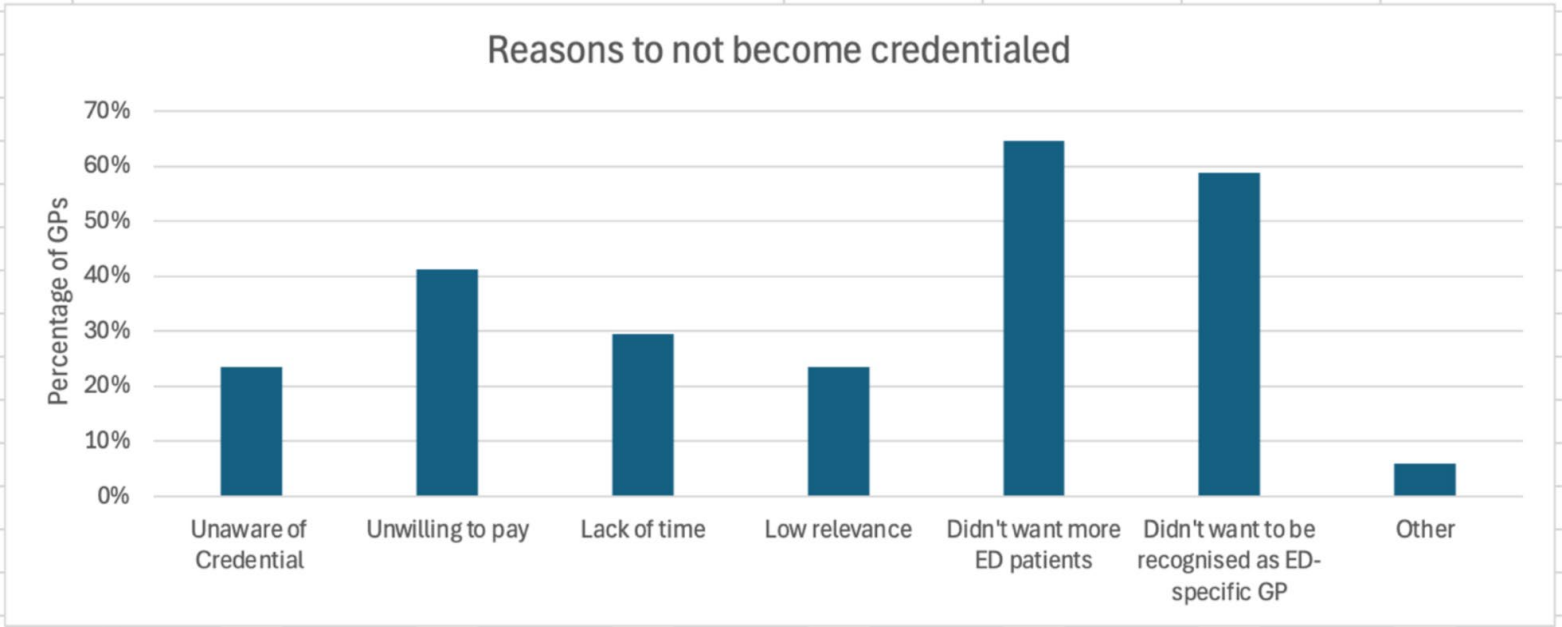
Reasons GPs provided for not becoming credentialed*. *Participants were able to select more than one answer

GPs were asked why they became credentialed. As shown in Fig. 1, most credentialed GPs reported that they were motivated to become credentialed to receive formal recognition of their ED training (n = 15, 71.4%) and just under half indicated their motivation was to improve their knowledge about EDs (n = 9, 42.9%). Considering why they did not become credentialed, non-credentialed GPs most commonly reported that they: (1) did not want to increase their ED patient caseload (n = 11, 64.7%); (2) did not want to be identified as an ED-specific GP (n = 10, 58.8%); and (3) were unwilling to pay for the Credential (n = 7, 41.2%) (see Fig. 2). Furthermore, 12 (70.6%) non-credentialed GPs reported that they did not think the Credential would benefit their practice.

Credentialed GPs also reported on changes to their practice after becoming credentialed. Three quarters (n = 16, 76.2%) of credentialed GPs indicated that the Credential had not changed the number of patients with an ED that they were seeing each week, whereas a small number of GPs indicated an increase in patient numbers (small increase n = 4 [19.0%]; moderate increase n = 1 [4.8%]).

Considering the processes involved in becoming credentialed, thirteen (61.9%) credentialed GPs rated the ease of applying for the Credential as fair (n = 8, 38.1%), easy or very easy (n = 5, 23.8%), whereas 8 (38.1%) credentialed GPs rated the application process to be difficult or very difficult (n = 8, 38.1%). Thirteen (61.9%) credentialed GPs had not created a profile in the directory, and 26 (68.4%) of all GPs had never used the directory, with similar proportions of credentialed GPs (n = 13, 61.9%) and non-Credentialed GPs (n = 13, 76.4%) having not used the directory.

### Qualitative analysis

Qualitative analyses explored free-text responses to questions asking about why credentialed GPs had not created a profile, potential benefits of the Credential to participants’ GP practice and motivations to become credentialed, as well as suggestions for improvement to the Credential and its systems. Two questions were asked of credentialed GPs (n = 21), three questions were asked of non-credentialed GPs (n = 17), and one question was asked of both credentialed and non-credentialed GPs (38) (see Supplemental information for full questions).

**Fig. 3:**
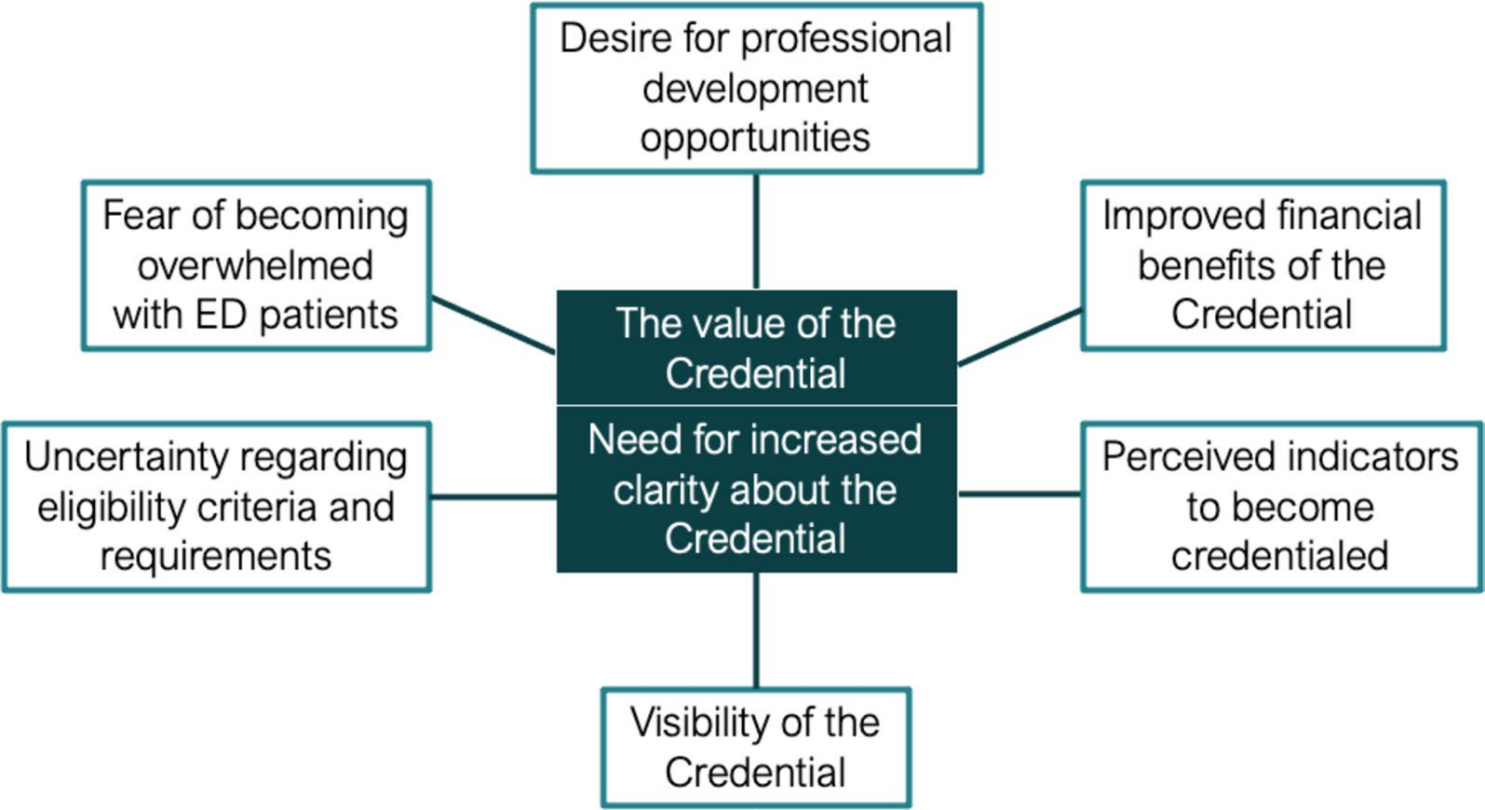
Thematic map of GPs’ perceptions of the ANZAED Eating Disorder Credential for GPs

Content analysis of GPs’ perceptions about the directory and Credential generated two themes: (i) the value of the Credential; and (ii) the need for increased clarity about the Credential. Each of these main themes had three subthemes as depicted in Fig. 3.

#### Theme 1: the value of the credential

The first theme provided insight into the inter-relationship between perceived burdens and benefits of the Credential present in the GP’s accounts.

- Improving financial benefits of the Credential

Many of the GPs talked about the lack of financial incentives for them to become credentialed in light of the costs of training. For example: *“There is no additional access to higher Medicare rebates, *etc*. for being credentialed, and thus it is not worth the cost”.*

(b) Fear of becoming overwhelmed with ED patients

The GPs also stated they were at full capacity, could not take on any more patients and talked about the added challenges in caring for patients with EDs: *“I would quickly be overwhelmed with eating disorder patients”.* Implicit in the sense of overwhelm is the likely complexities of ED care for GPs including medical impacts of EDs as well as psychological barriers that risk impeding recovery.

(c) Desire for professional development opportunities

Some of the GPs requested more professional development opportunities be included in the Credential, showing that they recognised the potential value of the Credential. For example, *“With the right training, my care is likely to be more effective and aligned with current best practice”.* While some GPs perceived a benefit of the Credential was demonstrating their commitment to best practice in the care of people with an ED, others noted barriers such as the financial cost and the potential for being credentialed increasing their current ED patient caseload.

#### Theme 2: need for increased clarity about the credential

The second theme identified some uncertainties about the requirements of the GP Credential and indicators need for credentialed GPs to appropriately screen for EDs, even if the patient is presenting reporting ED symptoms. Parallelling this need for clarity for indicators for GP credentialing in EDs perceived concerns of the invisibility of the Credential to GPs more broadly.

- Uncertainties regarding eligibility criteria and requirements

Some GPs’ responses indicated uncertainty about the differences between the requirements of the MHP and GP Credential. For example, the response of *“The cost and time required to achieve your expected supervision is unrealistic”* indicated that there was confusion about the requirements for the GP Credential that does not require ongoing supervision compared to the MHP Credential that does. It is possible, and likely, that uncertainties about the supervision requirements were a barrier for some GPs in attaining their profession specific Credential.

(b) Perceived indicator to becoming credentialed

Other GPs perceived EDs to impact narrower demographics, (e.g. young women) and hence did not perceive a need to be a credentialed GP based on their current patient demographics. Implicit in these perceptions are questions of health literacy, that is, EDs can impact people across genders, cultures and the lifespan, as well as the understanding that the Credential aims to support GPs and other health professionals working with all patient demographics.

(c) Visibility of the Credential

Some GPs recommended increasing promotion and/or stated they had previously been unaware of the Credential and connect·ed searchable directory: *“I didn’t know it existed”.* These ongoing uncertainties about the requirements, indicators and visibility of the GP Credential are likely barriers to the broader uptake of the GP Credential.

## Discussion

This study explored GPs’ experiences and perceptions of the Credential and identified perceived facilitators and barriers of the Credential. Reasons for GPs to become credentialed included receiving recognition for their training and improving their ED knowledge. However, many GPs thought the financial and workload limitations may have outweighed the benefit of being credentialed. Although some GPs stated that they chose not to become credentialed to avoid being identified as an ED-specific GP or increasing their ED caseload, most credentialed GPs did not perceive that becoming credentialed increased the number of ED patients they saw. Responses also indicated some misunderstanding of the aims and requirements of the Credential, and some responses indicated an absence of recognition of broad demographic of people who experience EDs and the need for GPs as first line health professionals to have skills and support in screening for EDs and in the provision of medical care. Nevertheless, there was a recognition of the importance of the Credential for GPs and health care professions and the need for the promotion of the Credential and directory to be improved.

Both credentialed and non-credentialed GPs described benefits of the Credential. The main reasons GPs became credentialed were for formal recognition of their training and to improve their knowledge about EDs. This suggests that they found merit in being credentialed, which was similarly found in previous research reporting on perceptions of dietitians and mental health professionals who were credentialed [20]. Some GPs also suggested increasing CPD opportunities that are included in the Credential including access to ED group supervision might further develop their professional skills. This interest shows that including ED supervision within or alongside the Credential may be well received by GPs and may increase the perceived value of the Credential. Such sentiments have been expressed previously in qualitative research in which credentialed dietitians and mental health professionals perceived that supervision helped strengthen their knowledge, competency and confidence working in the ED field, and helped them reflect on and debrief about their work, helping sustain themselves in their practice [23]. Similar benefits may also be received by GPs through supervision, which may have important implications in improving care for those with an ED.

Although some GPs identified benefits of the Credential, other GPs did not become credentialed as they did not want to be further involved with ED work. Many non-credentialed GPs did not want to increase their ED patient caseload (64.7%) or be identified as an ED-specific GP (58.8%) and expressed worry about this eventuating. This finding is consistent with previous research that has found that ED care providers feel a lack of competence [24] and have an increased risk of experiencing burnout [25], due to the complexities of their work [26]. Helping GPs to become more confident and knowledgeable in their care of ED patients may help them overcome fears of being overwhelmed. However, there are challenges in providing adequate ED education to GPs and healthcare professionals broadly due to insufficient educational resources and the limited availability of ED experts within tertiary education settings able to teach this content [27]. Hence, providing further training options might allow GPs to choose a more suitable and attainable education pathway. Although GPs may broadly benefit from greater training in ED management, it is acknowledged that not all GPs may want to become credentialed, and there may be challenges to overspecialisation in mental health [28]. ED management support tools for GPs (e.g. the freely available InsideOut Institute GP Hub) and enhancing university training for GPs are being explored to improve generalist care [12, 28]. However, the Credential for GPs was developed in response to immediate calls from people with an ED to improve their identification of and access to GPs who have knowledge and understanding in ED management. Moreover, interest in becoming credentialed by certain GPs is demonstrated by those already taking up the opportunity to become credentialed. The Credential could provide a package of additional professional development or supervision to help support GPs’ management of people with an ED. Moreover, individual or group supervision, in which GPs in the present study were favourable towards, could help improve their care and sustain them in their work, as shown in other studies focusing on supervision in allied health. Additionally, although non-credentialed GPs expressed concern that being credentialed would lead to an increase of ED patient caseload, credentialed GPs did not see such an increase in their caseload. This finding may also challenge this barrier to become credentialed and encourage more GPs to apply.

As with credentialed GPs, prior research found that credentialed dietitians and mental health professionals likewise did not observe an increase in their ED patient caseload during the Credential’s early implementation [20]. A possible explanation for the absence of change in patient numbers may be that most GPs had not created a profile, or personally used, the online searchable directory. However, many non-credentialed GPs stated they had a full patient load, so this workload limitation could be a reason for not creating a profile. Additionally, most non-credentialed GPs (64.7%) worried that the Credential might increase their visibility to help seekers, potentially deterring them from creating a profile in the directory if they became credentialed. Hence, although most GPs did not see an increase in their ED caseload, this is perhaps not something they were wanting to gain from being credentialed.

Some non-credentialed GPs thought the Credential was not worthwhile due to the financial cost and one subtheme showed that many credentialed GPs felt there should be improved financial benefits of the Credential. Existing research has identified cost and poor accessibility as barriers to becoming further qualified in mental health [29]. It also found that Medicare rebates, which are a payment from the Australian Government to cover certain health services, are an effective incentive to gaining additional qualifications in mental health [29]. Linking such rebates or other Medicare incentives to the provision of care by credentialed GPs may be a potential future strategy to reduce the perceived financial burden of becoming credentialed to GPs and increase GPs’ motivation to become and remain credentialed.

Our research has also revealed that at this early point in the GP Credential rollout, some ambiguity about the GP Credential persists. This includes some confusion regarding the eligibility criteria and requirements of the GP Credential. Some GPs referenced the longer training of the mental health professional Credential as a barrier, however seemed unaware of the less time intensive requirements of the GP Credential as an alternative. This misunderstanding may have discouraged GPs from becoming credentialed and could justify a need to provide clearer communication about the different ANZAED Eating Disorder Credentials. GPs may also have misconceptions that certain populations, such as elderly people, were unlikely to be affected by EDs, despite research showing that such populations are often mistakenly overlooked when presenting with ED symptoms [30]. Such stereotypes regarding who experiences EDs may have therefore led some GPs to feel that additional training and credentialing in EDs were not relevant to their patient caseload. Moreover, most GPs had never used the Credential’s searchable directory, thus missing potential benefits of identifying credentialed mental health professionals and dietitians for their patients. This further demonstrates that some GPs are not aware that the Credential could benefit their practice, as they may believe it is not relevant to them.

Interestingly, the study highlighted that there was insufficient visibility of the Credential, which could explain the uncertainties about the Credential. Improving the visibility of the Credential would create awareness about the requirements of the Credential and the impact it can have on people living with EDs. Additionally, the increased visibility and recognition may allow the Credential to reach new GPs, giving them the opportunity to become credentialed.

Strengths of the study include the use of mixed-methods design, which produced concise answers from the participants, whilst providing them the opportunity to expand on their answers with open-ended questions. Two authors met multiple times to review the qualitative data and inductively identify themes to stretch their respective interpretive lenses. Another strength was the approximately equal distribution between credentialed and non-credentialed GP participants, which allowed identification of differences in perspectives, and gained insight from both cohorts. A limitation of the study was the underrepresentation of GPs credentialed as MHPs, as they only made up 9.5% of credentialed participants in this study, whereas they form 21.6% of all credentialed GPs across both pathways open to GPs for credentialing. Furthermore, this study had an overall small sample size and there was an unequal gender and state distribution of participants, so the results may not necessarily reflect the views of certain states or the male GP population. In this study, it was also difficult to assess any change in ED patient caseload after becoming credentialed as data were collected from participants’ retrospective recall. Due to the recency of the Credential’s implementation, it is not likely well known by the general public and clinician network, limiting any observed effect on caseload. Also given that cost was consistently identified as a barrier during this study, there was also a lack of differentiation between bulk billing GPs, who provide free healthcare to patients, and private billing GPs, who charge patients for their services. For future financial strategies to target the right demographics, research diversifying and expanding the GP participant samples is needed to identify if there are varying perspectives for bulk billing and private billing GPs.

Overall, credentialed GPs valued the Credential in their work providing care to people with an ED. However, barriers included lack of clarity about the requirements for becoming and maintaining the Credential, and a reluctance to be identified as an ED specialist GP due to the potential of increasing their ED patient caseload. Although this study did not have the scope to explore the reason for GPs reluctance to be identified as specialising in the treatment of EDs, future research could explore this in more depth to understand ways to address these barriers for GPs, including through targeted CPD training and ongoing supervision. As the Credential continues to be rolled out, the perceived financial and workload limitations need to be addressed to incentivise GPs to undertake training and build confidence in ED care, helping to address the urgent need for early identification and treatment of EDs. For the successful utilisation and future sustainability of the GP Credential, this research highlights the importance of improving communication about the GP Credential to both GPs and help seekers, as well as increasing awareness amongst GPs that EDs do not discriminate by age, gender or cultural background and are relevant to every GP caseload.

## Supplementary Information

Additional file1.

## Data Availability

The datasets used and/or analysed during the current study are not publicly available. They may be available from the corresponding author upon reasonable request and in accordance with Human Research Ethics permissions. Permission for the data to be made publicly available is not sought as it would identify participants.

## Abbreviations

ANZAED: Australian and New Zealand Academy for Eating Disorders
GP: General practitioner
ED: Eating disorder
RACGP: Royal Australian College of General Practitioners
MHP: Mental health professional
CPD: Continuing professional development
EDTP: Eating Disorder Treatment Plan
AHPRA: Australian Health Practitioner Regulation Agency
