# Supplementary material for: General practitioner experiences and perceptions of the ANZAED Eating Disorder Credential
Source: J Eat Disord. 2026 May 19;13(Suppl 1):298. doi: 10.1186/s40337-026-01622-w (PMC13185159; doi:10.1186/s40337-026-01622-w)
Supplement: Supplementary file 1 — Additional file1. [file 40337_2026_1622_MOESM1_ESM.docx]

**Supplemental information: Qualitative questions asked in the survey**

|  | Question asked of credentialed or non-credentialed GPs | No. participants asked | No. responses |
| --- | --- | --- | --- |
| “Please tell us why you did not create a profile.”  *Only asked if they answered “No” to previous question “Did you set up a clinician profile on the ‘Find Eating Disorder Help’ directory?”. | Credentialed GPs | 21 | 13 |
| “Do you have any suggestions on how to improve the searchable directory?” | Credentialed and non-credentialed GPs | 38 | 9 |
| “Please provide reasons for your response.”  *Asked in response to previous question “Do you think the Credential would benefit your clinical practice?” | Non-credentialed GPs | 17 | 17 |
| “What would motivate you to become credentialed?” | Non-credentialed GPs | 17 | 13 |
| “Do you have any other recommendations on how the ANZAED Eating Disorder Credential for GPs could be improved?” | Credentialed and non-credentialed GPs | 38 | 23 |
